# Does prevalence of sexual dysfunction differ among the most common causes of infertility? A cross-sectional study

**DOI:** 10.1186/s12905-022-01708-y

**Published:** 2022-04-27

**Authors:** Mahnaz Ashrafi, Nadia Jahangiri, Shahideh Jahanian Sadatmahalleh, Negin Mirzaei, Naiiere Gharagozloo Hesari, Frahnaz Rostami, Seyedeh Saeedeh Mousavi, Mona Zeinaloo

**Affiliations:** 1 Department of Endocrinology and Female Infertility, Reproductive Biomedicine Research Center, Royan Institute for Reproductive Biomedicine, ACECR, Number 12, East Hafez Avenue, Banihashem Street, Resalat Highway, Tehran, Iran; 2 Department of Reproductive Health and Midwifery, Faculty of Medical Sciences, Tarbiat Modares University, Jalal Al-Ahmad Highway, Nasr Bridge, 14115-111 Tehran, Iran; 3 Department of Obstetrics and Gynecology, Faculty of Medicine, Iran University of Medical Science, Hemmat Exp. Way, Tehran, Iran; 4 Department of Midwifery and Reproductive Health, Iran University of Medical Sciences, Tehran, Iran; 5 Department of Community Medicine, Tehran University of Medical Sciences, Tehran, Iran

**Keywords:** Infertility, Female sexual function, Prevalence

## Abstract

**Background:**

Sexuality as a fundamental component of women’s health, can be affected by infertility. The current study aimed at comparing the prevalence of sexual dysfunction among women with the most common causes of infertility.

**Methods:**

The current cross-sectional study was conducted on 240 infertile females with infertility due to polycystic ovary syndrome (PCOS, n = 80), endometriosis (n = 80) and male factor (n = 80) at Royan Institute for Reproductive Biomedicine (Tehran, Iran) and 160 fertile women at health care centers, between May 2016 and June 2017. Sexual function was assessed by Female Sexual Function Index (FSFI). Data were analyzed using SPSS (version 25.00) and differences were regarded statistically significant at *p* < 0. 05.

**Results:**

The prevalence of female sexual dysfunction was 98.8% in women with PCOS, 100.0% in those with endometriosis, and 80.0% in those with male factor infertility. Overall, 36.2% of the enrolled fertile women were suffering from sexual dysfunction.

**Conclusions:**

There was an association between the prevalence of female sexual dysfunction or individual domain scores of the FSFI, and infertility etiologies. Therefore, infertility care providers are required to take this into consideration and develop preventive strategies in this regard.

**Plain summary:**

Infertility as a major health care problem affects an estimated 8–12% of couples of reproductive age globally and sexuality as an important part of women’s health, can be affected by infertility. In this study, the prevalence of sexual dysfunction among women with the most common causes of infertility has been evaluated.

The present study was conducted on 240 infertile females with infertility due to polycystic ovary syndrome (PCOS, n = 80), endometriosis (n = 80) and male factor (n = 80) at Royan Institute (Tehran, Iran) and 160 fertile women at health care centers, between May 2016 and June 2017. Sexual function was assessed by Female Sexual Function Index (FSFI); a brief self-report measure of sexual functioning.

Results highlight that the prevalence of sexual dysfunction in women with endometriosis and PCOS was higher than in other groups. As, the prevalence of female sexual dysfunction was 98.8% in women with PCOS, 100.0% in those with endometriosis, and 80.0% in those with male factor infertility. In total, 36.2% of the enrolled fertile women were suffering from sexual dysfunction.

The results point to an association between the prevalence of female sexual dysfunction and causes of infertility. Therefore, infertility care providers are required to take this into consideration and develop preventive strategies in this regard.

## Background

Infertility is one of the major health care problems that is of serious concern [1]. From different parts of the world, diverse prevalence and causes of infertility have been reported. This inconsistency is due to variations in environmental conditions influencing reproductive behaviors [2, 3]. It is estimated that globally 8–12% of couples of reproductive age suffer from infertility [4]. Besides couples for whom no known cause is identified (25%), males and females are found to be responsible for 30 and 45% of infertility cases, respectively [5]. A range of causes may produce infertility in women from which, endometriosis with a prevalence of 20–50% [6] and PCOS with a prevalence of 15–20% [7] are the two of the most-common causes.

Sexual function has a known impact on the quality of life [8] and when disturbed, cause difficulties in conception [9]. Sexual dysfunction is multifactorial in etiology and may include psychosocial, neurologic, and hormonal issues [10]; this may be either a consequence or a cause of infertility [1, 11]. Although sexual function is related to both partners, sexual disorders are more prevalent in women [8, 10, 12, 13] and according to studies, women with infertility have higher rates of sexual dysfunctions (5–55%) compared to the general population [1, 11, 14, 15]. Although extensive research has been conducted in the field of sexual dysfunction in different female groups in Iran, there is scarce information on this topic in infertile women [1]. Therefore, performing a study in this issue would help to develop strategies to improve life quality in infertile couples. Since no study has yet compared sexual function based on the different causes of infertility, the present work compared sexual dysfunction and its prevalence among Iranian females with three most-common infertility etiologies, using a validated questionnaire.

## Methods

### Design and data collection

In this cross-sectional study, we evaluated prevalence of sexual dysfunctions in 400 women admitted between May 2016 until June 2017, at Royan Institute and health care centers in Tehran, Iran. The participants were selected through convenient sampling method. The study was approved by the Institutional Review Board of the Royan Institute Research Center and the Royan Ethics Committee (IR.ACECR.ROYAN.REC.1395.97) and performed according to the Helsinki Declaration. Informed consent was obtained from all individuals enrolled. In the present study, participants were divided into two groups of fertile (n = 160) and infertile (n = 240) women. Fertile women were recruited from the health-care centers in Tehran and all had used a condom as a birth control method; infertile women were those with primary or secondary infertility and were grouped based on the existence of polycystic ovary syndrome (PCOS), endometriosis and male factor, as the most common causes of infertility.

The diagnosis of PCOS was according to the Rotterdam criteria [16]. Male factor infertility was determined by an abnormal semen analysis as established by World Health Organization (WHO) [17]. Endometriosis diagnosis was confirmed by laparoscopy. Based on laparoscopic findings, women with abnormalities other than endometriosis were excluded.

Inclusion criteria consisted of an age range of 18 to 45 years, living with the husband, and being sexually active in the last 4 weeks. Participants who filled the questionnaire incompletely or refused to complete the study were excluded.

### Measures

Socio-demographic and obstetric characteristics including women’s age, body mass index (BMI), educational level, occupational status, duration of infertility, and type of infertility, were recorded.

### Sexual function

Women’s sexual function in the last 4 weeks was measured using the Female Sexual Function Index (FSFI). This scale consists of 19 items and assesses six main aspects of sexual functions as follows: sexual desire, arousal, lubrication, orgasm, satisfaction, and pain.

Each question was rated on a scale from 0 or 1 to 5. The sum of each domains’ scores was multiplied by a certain factor. The overall scale score ranged from 2 to 36, with higher scores meaning better sexual function. In the Iranian population, the psychometric properties of the questionnaire were previously verified [18]. In this study, the Persian version of the FSFI questionnaire translated by Mohammadi et al. was used. Therefore, scores < 3.3 in desire, scores < 3.4 in arousal and orgasm, scores < 3.8 in satisfaction and pain, scores < 3.7 in lubrication and the total scores < 23 were considered to be FSD [19].

### Depression and anxiety

Hospital Anxiety and Depression Scale (HADS) questionnaire was used to evaluate the severity of anxiety and depression. This survey has 14 questions composed of two subscales that examined anxiety (7 items) and depression (7 items). Each item was rated on a 4-point Likert-type scale ranging from 0 to 3 (0 = never, 1 = seldom, 2 = sometimes and 3 = always) with a total score range of 0–21 for both subscales. Higher scores represent greater anxiety and depression state. The validity and reliability of this questionnaire were previously shown among Iranian population [20].

### Statistical analysis

Data were analyzed using Statistical Package for the Social Sciences (SPSS version 25.00). Kolmogorov–Smirnov test was used to analyze normality for continuous variables. Data were compared by Chi-square or One-Way ANOVA test followed by the Tukey post hoc test if appropriate. Results are reported as mean ± SD or number (percentage). A *P* < 0.05 was considered statistically significant.

## Results

**Fig. 1:**
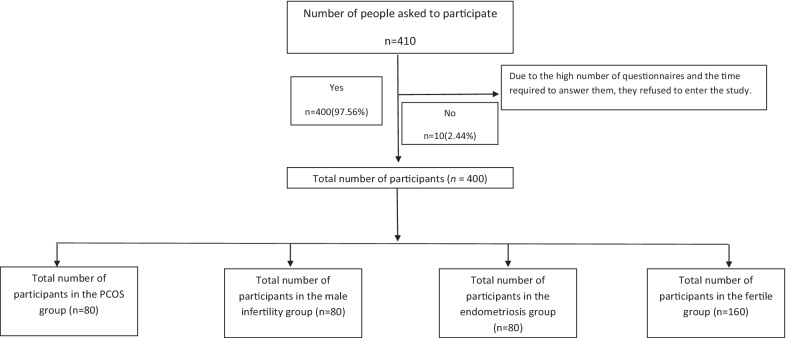
Flow chart of the study

Among the 410 participants interviewed for this study, ten women didn’t meet the inclusion criteria (2.44%) (Fig. 1). Table 1 describes the socio-demographic and obstetric characteristics of the participants. It illustratesthere were statistically significant differences between the study groups in terms of age, BMI, occupational status, educational level, and type of infertility (*P *˂ 0.05). Also, no illiterate person participated in the study and most of them were housewives. The majority of subjects in the case group (67.5%) suffered from primary infertility and 32.5% of infertile women were affected by secondary infertility (*P* = 0.006).The two groups were comparable with regard to duration of infertility. There were no significant differences between the mean total score of depression and anxiety and total HADS in PCOS, male factor, endometriosis, and fertile women.

**Table 1:** Comparison of socio-demographic and obstetric characteristics of participants

Variables	PCOS N = 80	Male factor N = 80	Endometriosis N = 80	Fertile N = 160	*P* value
Age (years)*	31.94 ± 4.44	32.41 ± 5.70	28.28 ± 4.30	31.66 ± 1.89	˂0.001
Education**					
Under Diploma	9(11.3)	1(1.3)	24(30.0)	1(0.6)	˂0.001
Diploma	65(81.3)	32(40.0)	33(41.3)	70(43.8)	
University	6(7.5)	47(58.8)	23(28.7)	89(55.6)	
Job**					
Housewife	75(93.8)	51(63.7)	42(52.5)	117(73.1)	˂0.001
Employ	5(6.3)	29(36.3)	38(47.5)	43(26.9)	
BMI*	27.04 ± 3.24	26.76 ± 4.43	25.49 ± 3.80	26.13 ± 3.75	0.046
Duration of infertility	2.93 ± 2.03	2.96 ± 2.38	2.80 ± 2.02	–	0.880
Type of infertility					
Primary	43(53.8)	60(75)	59(73.8)	–	0.006
Secondary	37(46.3)	20(25)	21(26.3)	–	
HADS					
Anxiety	7.0 ± 4.2	7.5 ± 5.0	7.0 ± 4.4	6.3 ± 4.0	0.184
Depression	4.5 ± 3.5	5.3 ± 4.7	5.8 ± 4.3	4.8 ± 3.9	0.187
Total scores	11.5 ± 6.9	12.7 ± 9.1	12.7 ± 7.9	11.0 ± 7.2	0.259

PCOS, polycystic ovary syndrome; BMI, Body Mass Index; HADS, Hospital Anxiety and Depression Scale

*Values are given as mean ± SD and compared using One-way ANOVA

**Values are given as number (%) and compared using Chi-squared test

The comparison of various aspects of sexual dysfunction are shown in Table 2. As scan be seen in Table 4, there was a statistically significant difference in the scores of different dimensions of sexual dysfunction between the two groups (*P* ˂ 0.001). The score of sexual dysfunction in terms of desire, arousal, lubrication and total FSFI was lower in women with PCOS than other groups and women with endometriosis had lower scores in total orgasm, satisfaction, and pain. Furthermore, fertile women achieved the highest score in all dimensions. There was a statistically significant difference between the two groups in all aspects of sexual function (*P *˂ 0.001); however, for pain no significant difference was found between fertile women and those with male factor infertility.

**Table 2:** Comparison of different aspects of sexual dysfunction in the study groups

Variable	PCOS N = 80	Male factor N = 80	Endometriosis N = 80	Fertile N = 160	*P* value*
Desire	3.04 ± 0.74	4.11 ± 0.77	3.78 ± 0.70	4.39 ± 1.14	˂0.001^a^
Arousal	3.21 ± 0.75	4.20 ± 0.77	3.88 ± 1.07	5.03 ± 1.05	˂0.001 ^a^
Lubrication	3.52 ± 0.80	4.61 ± 0.79	4.29 ± 0.61	4.99 ± 0.83	˂0.001 ^a^
Orgasm	3.50 ± 0.69	3.72 ± 0.77	3.00 ± 0.77	4.49 ± 0.81	˂0.001 ^a^
Satisfaction	4.16 ± 0.95	4.54 ± 0.79	3.81 ± 0.73	5.10 ± 0.78	˂0.001 ^a^
Pain	4.16 ± 1.22	4.96 ± 0.31	3.68 ± 0.74	5.05 ± 0.85	˂0.001 ^b^
FSFI	21.60 ± 2.90	26.16 ± 1.78	22.46 ± 1.84	29.07 ± 2.50	˂0.001 ^a^

PCOS, polycystic ovary syndrome; FSFI, Female Sexual Function Index

*Values are given as mean ± SD and compared using One-way ANOVA

^a^*p* < 0.05 between two groups as assessed by Tukey’s post hoc pairwise comparisons following ANOVA

^b^*p* < 0.05 between two groups as assessed by Tukey’s post hoc pairwise comparisons following ANOVA except between Male factor and fertile groups

Table 3 presents the prevalence of female sexual dysfunction (FSD) between participants. From this data, we can see the significant differences in the prevalence of FSD among groups (*P* ˂ 0.001) and 98.8% of women with PCOS (n = 79), 80.0% of infertile women with male factor = (n = 64), and 100.0% of women with endometriosis (n = 80) are suffered from FSD. There was a significant difference in the prevalence of sexual dysfunction among FSFI subgroups and total scores among the four groups. Prevalence of sexual dysfunction in women with endometriosis and PCOS was higher than other groups, except for orgasm and pain which were more common in women with endometriosis.

**Table 3:** Comparison of prevalence of sexual dysfunction in different study groups

Variable	PCOS N = 80	Male factor N = 80	Endometriosis N = 80	Fertile N = 160	*P* value
Desire	41(51.2)	10(12.5)	15(18.8)	26(16.3)	˂0.001
Arousal	46(57.5)	9(11.2)	27(33.8)	15(9.4)	˂0.001
Lubrication	54(67.5)	17(21.2)	14(17.5)	14(8.8)	˂0.001
Orgasm	38(47.5)	26(32.5)	56(71.8)	9(5.6)	˂0.001
Satisfaction	27(33.8)	5(6.2)	25(31.6)	5(3.1)	˂0.001
Pain	31(38.8)	0(0.0)	38(47.5)	21(13.1)	˂0.001
FSD	79(98.8)	64(80.0)	80(100.0)	58(36.2)	˂0.001

PCOS, polycystic ovary syndrome; FSD, female sexual dysfunction

*Values are given as a number (%) as assessed by Chi-squared test

Table 4 provides an overview of FSFI scores in women with primary and secondary infertility. There were 162 females with primary infertility, and 78 with secondary infertility. The prevalence of FSD was 92.0% (n = 149) and 94.9% (n = 74) in primary and secondary infertile females, respectively, which was not significantly different (*P* = 0.413, Table 4). However, there was a statistically significant difference between primary and secondary infertility groups in some aspects of sexual function including arousal, lubrication, and satisfaction (*P *˂ 0.05).

**Table 4:** Comparison of FSFI scores in primary and secondary infertile women

	Primary infertility N = 162	Secondary infertility N = 78	*P* value
Desire*	3.65 ± 0.89	3.62 ± 0.79	0.762
Arousal*	3.68 ± 0.98	3.95 ± 0.91	0.039
Lubrication*	4.22 ± 0.87	3.98 ± 0.84	0.046
Orgasm*	3.36 ± 0.82	3.50 ± 0.75	0.225
Satisfaction*	4.01 ± 0.89	4.50 ± 0.76	˂0.001
Pain*	4.23 ± 1.01	4.34 ± 0.97	0.440
FSFI*	23.17 ± 3.23	23.90 ± 2.31	0.073
FSD**	149 (92.0)	74(94.9)	0.413

PCOS, polycystic ovary syndrome; FSFI, Female Sexual Function Index; FSD, female sexual dysfunction

*Values are given as mean ± SD as assessed by One-way ANOVA

**Values are given as a number (%) as assessed by Chi-squared test

Table 5 provides the results of analysis of covariance to compare FSFI score in study groups and it showed that the average score of FSFI in infertile women with PCOS, male factor, and endometriosis was 7.47, 2.91, and 6.59 lower than fertile group, respectively.

**Table 5:** Analysis of covariance (ANCOVA) to study total FSFI score on study groups

Variables	B	Standard error	t	*P* value*
Age	0.10	0.030	0.320	0.749
BMI	− 0.005	0.031	− 0.160	0.873
PCOS	− 7.466	0.324	− 23.074	˂0.001
Male factor	− 2.914	0.324	− 9.008	˂0.001
Endometriosis	− 6.586	0.338	− 19.483	˂0.001
Fertile	0^a^			

Dependent variable: total FSFI

^a^This parameter is set to zero because it is redundant

*ANCOVA

## Discussion

To the best of our knowledge, this is the first study to compare the prevalence of sexual dysfunction among women suffering from most-common causes of infertility, including PCOS, endometriosis and male factor. Our study demonstrated that:

1. The lowest overall FSFI score was for women with PCOS and the most common problems in these women were sexual desire, arousal, and lubrication. Previous studies showed controversial results on sexual function in women with PCOS. Fliegner et al. [21] studied sexual function and socio-sexual difficulties in women with PCOS and found that these women were no different from those of the control group in terms of the number of sexual disorders, but their overall FSFI score was much lower than the control group. It seems that women with PCOS feel embarrassed and limit themselves because of hirsutism and obesity. In fact, these women feel unattractive due to hirsutism and their acne, and they limit themselves even more in the society. These women are not self-sufficient and usually do not feel good about their bodies, so this inner feeling can affect the physiological function of the body and directly affect the process of sexual desire and arousal [22, 23]. We also realized for these women, the most common problems were with desire, arousal and lubrication, which can be related to the internal sense of self, infertility and even taking medications like contraceptives for reducing acne, hirsutism and regulating the menstrual cycle [24], and also anti-androgens and GnRH antagonists used during infertility treatment [25–29]. Our findings are inconsistent with the study conducted by Battaglia et al. [30] who found that in PCOS women, probably, moderate hirsutism and hyperandrogenism do not induce the sense of loss of feminine identity and have no impact on sexual self-worth and sexual satisfaction. This difference is probably due to differences in the measurement tools. The tools used by Battaglia et al. in their study, were the male sexual fantasy questionnaire (MSFQ) and Beck’s Depression Inventory Questionnaire (BD).
2. In women with endometriosis, the overall score of orgasm, satisfaction, and pain was lower than the other groups. Consistent with our work, Tripoli et al. [31] studied quality of life and sexual satisfaction in women suffering from chronic pelvic pain (CPP) with or without endometriosis. They found that CPP caused by endometriosis or other gynecological conditions, leads to a significant reduction of quality of life and sexual satisfaction. In their study, 40% of those suffering from endometriosis or CPP caused by other gynecological diseases, were sexually unsatisfied. They reported a decreased frequency of sexual intercourse and vaginismus, and aversion, and reduced expression of sensuality. Giulia et al. [32] evaluated sexual satisfaction, desire, and orgasm, and pelvic problem interfering with sex in women with deep infiltrating endometriosis. The results of their study showed that women with endometriosis have sexual dysfunction that is associated with reduced quality of life. They also found that the presence of dyspareunia in these women led to a decrease in sexual desire and thus sexual dysfunction. One of the most common complaints of women with endometriosis is pain. Depending on the severity of the pain, women's daily activities, sexual activity, and even entertainment were reduced. Also, these limitations caused mood swings, irritability and sexual dysfunction in a person with a sexual partner [32]. In fact, in these women, the issue of dyspareunia and pain during sex and orgasm led to a decrease in sexual desire and the number of times it occurs; thus, affecting the level of sexual satisfaction. Verit et al. [33] found similar results and reported that 69.9% of women with chronic pelvic pain had sexual dysfunction. During sexual intercourse, these women thought that they would feel pain, which caused them anxiety.
3. The results of this study indicate that the prevalence of FSD in women suffering from male infertility is lower than women with endometriosis and PCOS, however, it is still significantly higher than the fertile group. Infertility has deleterious effects on the sexual relationships of a couple, also male and female partners cannot be deemed as separate [34]. Hence, when they are diagnosed as infertile, their self-identity will be threatened and both of them feel less attractive as not only femineity is associated with maternity and fecundity, also male virility is linked with the ability to impregnate a female [35]. However, the effects of male infertility on FSD have not been identified completely. In the light of the importance of sexuality in couple’s relationship and neglected effects of male infertility on FSD, quantifying sexual strain and identifying vulnerable subjects should be considered in future research to design specific interventions in order to promote the couple’s sexual functioning [34].
4. As a final result, we found that the prevalence of sexual dysfunction in the primary infertility group was not significantly different from the secondary infertility group in all subscale except arousal, lubrication and satisfaction (*p* < 0.05). In our study, sexual arousal and satisfaction were significantly lower and lubrication was higher in women with primary infertility compared to women with secondary infertility. It appears that problems with arousal and satisfaction are the main causes of sexual problems in women with primary infertility. This may be due to the fact that the level of stress is higher in women with primary infertility [36], and women with secondary infertility might be probably more comfortable with having children and more likely to have sex. It may be hypothesized from this finding that the stress produced by infertility impacts the psychological or relationship aspect of sexuality (arousal) more than the physical aspects (lubrication), but this cannot be conclusively stated based on these data [10]. Although in our study, there was no significant difference between the mean total score of anxiety (7.05 ± 4.64 vs. 6.77 ± 3.95; *P* = 0.761), depression (5.41 ± 4.49 vs. 4.32 ± 3.4; *P* = 0.211) and total HADS (12.39 ± 8.43 vs. 11.09 ± 6.81; *P* = 0.430) between the two groups of primary and secondary infertility, respectively, the mean score was slightly higher in women with primary infertility than those with secondary infertility; this small difference might be clinically important and might have a potential effect on some aspects of sexual function.

The results of various studies in this regard are inconsistent. Keskin et al. [10] evaluated prevalence of sexual dysfunction between primary and secondary infertile women. They found a higher prevalence of sexual dysfunction in secondary infertile women in comparison with primary infertile women. In this study, secondary infertile women had decreased sexual desire, orgasm, and satisfaction compared to the primary group. But in the study conducted by Shahraki et al. [37] in Iranian women with infertility, different results were shown. They found that sexual dysfunction was more common in women with primary infertility. Also, their results showed that total FSFI score and all of its domains were significantly higher in controls vs. infertile ones and only desire subscale was significantly higher in the primary group. In our study, except for satisfaction, in other subscale of sexual function, no significant difference was found between the two groups.

In societies such as Iran, according to the cultural factors rooted and penetrated in individuals’ deep unconscious layers, women’s feelings of satisfaction, perfection, and value are firmly dependent on their fertility and motherhood ability. Thus, It is undeniable that infertility has detrimental effects on quality of life and marital satisfaction [38]. Infertile women are at a higher risk of sexual dysfunction compared to fertile women and it is revealed that more than 60 percent of Iranian infertile women are suffering from sexual dysfunction [39]. Moreover, mentioning and assessing sexual dysfunction have been neglected in this mentioned population by healthcare services. This study highlighted the urgent need for further comprehensive policies for assessing and intervening with sexual dysfunction in various causes of infertility to enhance and improve their sexual quality of life. In spite of the importance of the present results, this study has some limitations. The main limitations of our study are external validity or the generalizability of the study due to the limited number of subjects involved, the multifactorial nature of female sexual dysfunction and data collected from Iranian women.

## Conclusions

There was an association between the prevalence of female sexual dysfunction or individual domain scores of the FSFI and causes of infertility. The prevalence of sexual dysfunction is high in infertile women with endometriosis and PCOS. Therefore, infertility care providers are required to take this into consideration and develop preventive strategies for this issue.

## Data Availability

The datasets used and/or analyzed during the current study are available from the corresponding author on reasonable request.

## Abbreviations

PCOS: Polycystic ovary syndrome
FSFI: Female Sexual Function Index
HADS: Hospital Anxiety and Depression Scale
FSD: Female sexual dysfunction
MSFQ: Male sexual fantasy questionnaire
BD: Beck’s Depression Inventory Questionnaire
CPP: Chronic pelvic pain
ANCOVA: Analysis of covariance
